# The heavy weight of COVID leadership: an analysis of one international school’s leadership during the pandemic

**DOI:** 10.1177/14752409231157635

**Published:** 2023-04-05

**Authors:** Sarah Pearce

**Affiliations:** University of Bath, UK

**Keywords:** Leadership and management, transactional and transformational leadership, educational leadership, international schools, pandemic leadership

## Abstract

The COVID-19 pandemic has created unprecedented chaos all over the world, and schools and their leaders have not escaped its impacts. This article analyses the leadership actions of the team from one international school in reopening after mandated lockdown; it seeks to contribute to the discourse on school leadership during these unprecedented times and to share leadership lessons with those bearing the weight of responsibility of leadership during the pandemic. Using the lens of a framework drawn from the example of Prime Minister Jacinda Ardern’s leadership of New Zealand during the crisis, the actions of the school leaders are examined and linked to each of the good practices outlined. To connect this firmly to school leadership, the actions are categorised as either educational management or educational leadership, and then assessed to determine to what extent transactional and/or transformational leadership was appropriate to how the leaders responded to the various issues that arose during the events. From the analysis, I conclude that the actions of school leaders should fall under the guise of both leadership and management in order to successfully take a school through a period of uncertainty such as this, and should utilise both transformational and transactional leadership, dependent upon the circumstances with which they are faced.

## Introduction

‘Heavy is the head that wears the crown in the COVID era’

(Ferguson, 2020)

Whilst there is much research into positive or negative, effective or ineffective, practices of leadership and management, both inside and outside of education, the current crisis that the world faces has thrown many of these theories, germane in ‘normal times’, into disarray; it seems we are in a ‘crisis of leadership’ (Tourish, 2020: 261).

This article seeks to contribute to a discourse on school leadership in times of unprecedented crisis, when the challenge has not previously been faced anywhere in the world, through analysis of a vignette: reopening a school after months of mandated lockdown as a result of the coronavirus pandemic. Every day, schools face demanding situations, including economic, physical and political, but the COVID-19 crisis has forced school leaders to make decisions of health and safety, amongst a variety of other aspects of schools, that are not learned in leadership courses or postgraduate degrees; educational leaders have had to dig deep, rely on ever-changing scientific and medical advice, and make life-and-death decisions for the students and staff in their care. As Bush states in his recent (during COVID times) editorial for the Educational Management Administration & Leadership journal, ‘[e]ducational leadership should always be focused mainly on the needs of the children and young people, and addressing those needs has never been more important or more difficult’ (2020: 959).

In order to analyse this vignette, and to avoid a simple chronological description of events and actions, it is essential to select a theoretical lens through which to view the events. Given the unprecedented nature of the global pandemic, I felt it important to use a lens specific to the crisis. ‘While lessons of leadership will likely emanate from the pandemic for months and years ahead’ (Gedro et al, 2020: 395), I turned to a recent contribution from Wilson (2020), who analysed the pandemic response of Jacinda Ardern, Prime Minister of New Zealand, before creating ‘a framework of key practices that the case of New Zealand seems to indicate can be helpful for leadership in a pandemic context’ (p280). Given ‘the potentially life-altering consequences of good or poor leadership’ (Wilson, 2020: 280) in this situation, Ardern’s response is widely considered to have been one of the most effective in the world, giving her the ‘perceived status as the world’s lone conqueror of coronavirus’ (Smith, 2020); whether this perceived status is deserved, it is clear that the New Zealand response, under Jacinda Ardern’s leadership, has been successful and the framework that Wilson has devised is a useful tool in the analysis of leadership in a COVID-19 world.

In an effort to link the analysis more securely to the area of education, Wilson’s framework will be used in conjunction with Connolly, James and Fertig’s work on educational management, educational leadership and educational responsibility (2019). With their insistence that educational management and educational leadership are ‘categorically different’ (p505), this analysis will determine when each has been employed and with what level of success, judged according to the Wilson framework.

Further to this, and in line with Connolly et al (2019), I will delve into the educational leadership practices and review them in terms of transactional and transformational leadership, and how at times it was necessary to ‘call up people’s inner motivation to work on an intrinsically motivating task’ and, at others, to rely on ‘external stimulus’ (p513). Whilst Bass (2000) stated that ‘the future educational leaders of learning organizations will be transformational’, there were certainly times during this period that transactional leadership was necessary for the functioning of the organization; Avci (2015: 2760) describes transactional leadership as being uninterested in ‘innovative aspects of the employees’ as well as ensuring ‘activities keep going within the frame of fundamental mission and vision of the organization’.

In this article, the Wilson (2020) framework will be used to dissect the actions of leaders of one particular school, referred to here as School X, helping to determine the success of each action and of the overall success of the school’s reopening. Further to this, each action will be categorised as educational leadership or management, to demonstrate their differences, and finally the analysis will determine the extent to which transactional and/or transformational leadership was appropriate to how the leaders of School X responded to the various issues that arose during the vignette.

## School Context and Background to Vignette

School X is an international primary school located in a large city (City Z) in the United States; the school caters to students from 15 months to 11 years old. It is an international school, though it has a singular, national affiliation and is part of a larger network of for-profit schools across the world; by one currently recognised typology, it is a ‘“Type C” “non-traditional” international school’ (Hayden and Thompson, 2013: 5). Whilst being an international school, it caters to a very large population of local students (around 87%), the remainder of the 550 students being made up of the children of expatriate workers from many parts of the world. It is in an upmarket neighbourhood in the city; the majority of parents are tertiary-educated and are working professionals. The school fees are relatively high, though not the highest within the competitor group, and the parent body forms an active and vocal community.

The school has approximately 100 staff, both academic and administrative, with the Senior Leadership Team (SLT) made up of the principal, three assistant principals (including myself), the Director of Marketing and Admissions, and the Business Manager. Whilst there was consultation across the full SLT during the reopening, this vignette will focus on the work of the academic SLT (principal and assistant principals).

The vignette begins with planning for reopening after 13 weeks of mandated ‘stay-at-home order’ which began on 20 March 2020 (Petrella et al, 2020), no expected time-frame having been given upon closure. After a great deal of uncertainty, the lockdown extended until the summer break began at the end of June. At that time, no guidance had been given about how, or indeed whether, schools were to open after the summer. Conflicting information was a regular feature in the media, particularly around state *vs* local mandates (Leone, 2020) for schools. SLT were of the view that school should reopen, as safely as possible, after the summer break; it was also clear from the managers of the school group that unless forced to close through government mandate, the school would reopen after the summer break. This vignette represents the series of decisions and events that occurred throughout the summer in leading to reopening the school, as well as reflections on those decisions, and the changes that took place after reopening.

## Literature Review

### The Wilson (2020) Framework

As already noted, a global pandemic is a crisis rarely seen in modern history; as a result, ‘COVID-19 plunged educational leaders into an unprecedented calamity’ (Marshall et al, 2020: 30). In all industries, leaders and managers were called to make decisions that sought to protect not only businesses and organisations, but also lives, while examples of sometimes questionable and at other times excellent leadership played out in real time across the world.

Wilson (2020: 280) suggests that ‘leadership scholars have a useful role to play in both exposing bad leadership and highlighting good leadership’; Farhan agrees, stating that ‘[i]n order to improve leadership effectiveness, it is critical to highlight leadership approaches and practices that contribute to improving situations of uncertainty and instability’ (2021: 1). During the pandemic, a number of frameworks were created to guide leaders during a crisis both within and beyond education. These include analysis of government leadership (see Farhan, 2021; Haslam et al, 2021) as well as educational leadership (see Harris and Jones, 2020; Hulme et al, 2021; Marshall et al, 2020; Urick et al, 2021). Whilst any of these frameworks would have proved useful in analysing the vignette, as Wilson’s was published early it was therefore accessible during the planning stage of this article and, perhaps more importantly, the leadership and success of Prime Minister (PM) Ardern had resonated with me during the crisis. As Wilson (2020: 280) reflected on her personal experiences, I also did; I was touched personally by the experience of poor leadership in different contexts, and saw PM Ardern as a beacon of light, noting the difference she was making in New Zealand and, through scrutiny of our actions, wanted to determine the impact we, as leaders in our school community, were or were not making.

**Figure 1.**
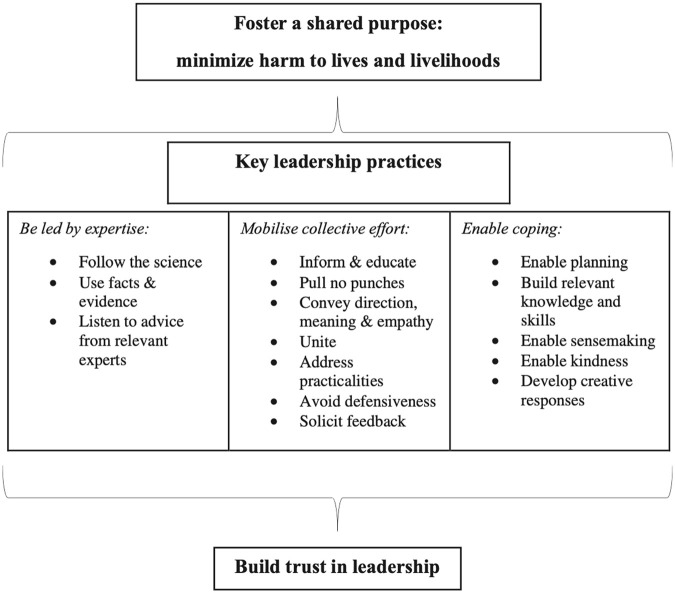
Wilson’s pandemic leadership: a good practice framework (Wilson, 2020: 285).

In her 2016 book, Wilson aimed to create a ‘flexible framework which can be used to invent forms of leadership uniquely tailored to specific circumstances and reflecting on different norms, values and assumptions’ (2016: 10). Her subsequent 2020 framework (see Figure 1) is an example of this invention; by trying to understand PM Ardern’s leadership through the ‘specific circumstances’ of the pandemic, she has highlighted the key practices that ‘the case of New Zealand seems to indicate can be helpful for leadership in a pandemic context’ (2020: 280). Using this framework, the actions of the leadership team in reopening the school will be evaluated.

### Educational Management, Educational Leadership and Educational Responsibility

Before moving into the vignette analysis using Wilson’s framework it is important, in order to make a clear link to education, to understand and define the difference between educational management and educational leadership; this understanding will help to determine which was required in each action when reopening the school.

Many a leadership trainer has defined the difference between ‘leadership’ and ‘management’, often suggesting one is more favourable than the other; management is about tasks, it is suggested, while leadership is about people:

‘All too often, people confuse leadership and management. Many believe managers have staff, whilst leaders have followers. Both require similar qualities, including technical and interpersonal acumen, but these are not synonymous.’

(Lush, 2019: 20)

**Figure 2.**
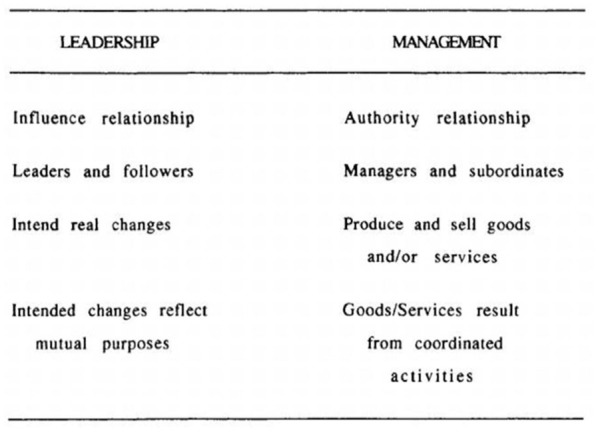
Distinguishing Leadership from Management (Rost and Burns, 1993: 149).

Rost and Burns (1993) agree with the assertion that leadership and management are not synonymous; they proposed the framework shown in Figure 2 to ‘make a clear separation between the two concepts’ (1993: 130).

Rost and Burns suggest that leadership involves influence, creating change and defining a vision, whereas managers have subordinates to ensure that tasks are completed; as Bass states, managers ‘engage in a transaction with their employees’ (1990: 19). These distinctions are supported, from an educational perspective, by Connolly et al (2019). Educational management, they argue, ‘is often used in relation to an organisational hierarchy’, with those higher up holding power and control over employees lower down (2019: 506); it is portrayed as focusing ‘on efficiency at the expense of institutional aims and purposes’ (2019: 506). In contrast, they propose that educational leadership is ‘the practice of influencing others to achieve goals in an educational context’ (2019: 510). Rost and Burns note that the difference between leadership and management is ‘a distinction between voluntary acceptance of another’s influence, on the one hand, and coerced compliance, on the other’ (1993: 131). Cuban (in Bush, 2007) makes the distinction that leadership encompasses ‘influencing others’ actions in achieving desirable ends’, whilst management consists of ‘maintaining efficiently and effectively current organizational arrangements’ (p8). Leadership is argued to be multidirectional (leaders to followers and vice versa), whereas management is unidirectional, or top down (Rost and Burns, 1993: 150); Bush concurs, describing the ‘normative view that leadership is more “acceptable” than management, with its bureaucratic “top-down” connotations’ (2019: 501).

Bush’s notion that leadership is ‘acceptable’ while management is not, is certainly not held by all leadership scholars. Rost and Burns very clearly reject this view (1993: 144), as do Connolly et al, suggesting that educational management is ‘neglected [and] downplayed’, while noting that there is ‘disregard of educational management’ in research, and in descriptions of schools and other educational institutions, that ‘underplays its importance in organising schools and colleges’ (2019: 505). Arguably, both management and leadership are necessary in order for educational institutions (or other organizations) to function effectively; as Cuban states, ‘I prize both managing and leading and attach no special value to either since different settings and times call for varied responses’ (in Bush, 2007: 8).

Connolly et al expand upon educational leadership and management with the concept of educational responsibility, ‘an internal sense of obligation’ which can be an action in itself, or used to support actions (2019: 513). Educational responsibility, they argue, underpins management as it ‘necessitates a designated individual carrying the responsibility for the functioning of a system in which others participate’; in leadership, individuals are responsible for their own influencing actions ‘regardless of whether they carry the responsibility for the functioning of a system in which they are influencing’ (2019: 514). Influence without responsibility can be a dangerous power, in which influence is rained on colleagues with abandon, without concern for the ‘responsibility for the functioning of the system’ (2019: 515). Such leaders can be held accountable, though they are not always, often due to their lack of official ‘leadership position’ within the school; it would be hoped that such influence is ‘undertaken responsibly’ (2019: 514).

The distinction between educational leadership and educational management is an important one, as is the understanding that one is not more important or useful than, or preferable to, the other; they are two sides of the ‘organisation of education institutions’ (Connolly et al, 2019: 504) coin, and should be viewed as such. This article will seek to determine when each was used throughout the vignette, with reference to the Wilson (2020) framework.

### Transactional versus transformational leadership

In linking with their work on leadership and influence, Connolly et al describe the types of motivation that move people to action: extrinsic and intrinsic (2019: 512), and link this to Bass’ (1990) transactional and transformational leadership theory. Berkovich (2018) defines transformational leadership as ‘aimed at changing followers’ beliefs, values and capabilities to promote their inclination to act beyond their self-interest, for the benefit of the organization’ (p349), linking to intrinsic motivation; in a study of Danish teachers, for instance, ‘findings show a positive correlation between transformational leadership and intrinsic motivation’ (Jensen and Bro, 2018: 544). Transactional leadership, meanwhile, is ‘generally associated with task management orientation and leader-subordinate exchange’ (Berkovich, 2018: 349), along with the promise of reward or threat of punishment for task completion or non-completion. As Bass effectively summarises, a transformational leader ‘emphasizes what you can do for your country’, whilst the transactional leader concentrates on ‘what your country can do for you’ (1999: 9).

Bass believes that transactional leadership ‘can be a prescription for mediocrity’ (1990: 20) and in much of his work espouses the virtues of leadership that is transformational rather than transactional. He contends that transformational leaders form better relationships and elicit greater contribution from their staff, and that they can make the difference between the success and failure of an organization (Bass, 1990). Berkovich (2018) supports this view with meta-analysis that shows transformational leadership has ‘a strong positive correlation with perceived leadership effectiveness’, as opposed to low positive correlation between perceived leadership effectiveness and transactional leadership (2018: 349). Connolly et al also acknowledge that ‘transformational leadership has been widely advocated as an appropriate model of educational leadership’ (2019: 513).

In much of the leadership literature there is a clear preference for transformational leadership. Analysis in this article will seek to establish whether and how transformational leadership was in evidence during the events of the vignette, whether transactional leadership was used at all, and the appositeness of these leadership choices, in light of the Wilson (2020) framework.

### Criteria for the analysis

Leithwood et al (2020) pose an interesting question for school leaders:

Under these conditions, what should I do?

(Leithwood et al, 2020: 10)

Throughout the events of the vignette, this question was asked in various forms, not only of ourselves as the SLT but by others. The SLT is experienced and, pre-pandemic, could have eloquently described leadership styles, their use and their effectiveness in leading School X; we knew ‘what to do’ in most circumstances. However, the global pandemic exhausted, decimated and demoralised everything we knew about leadership of a successful school; in fact, one could argue that more generally it redefined ‘success’ for schools.

Ultimately, this article seeks to evaluate the success of the leadership choices of the School X leadership team in reopening the school after lockdown. Guided by Wilson’s (2020) framework, the actions of SLT will be compared to the good practices outlined in that framework, with success determined by each individual practice, as well as the overall aim, which is to build trust in leadership. In order to link to the education context, these actions will be analysed using Connolly et al (2019) as either educational management or educational leadership, seeking to demonstrate the importance of each in leading a school through challenging circumstances. They will then be reviewed in terms of whether transformational or transactional leadership was necessary and/or successful at each step.

## Vignette and Analysis

For the purposes of this analysis, each element of the framework (as in Figure 1) will first be illustrated by examples from the school reopening, and then linked to the leadership concepts discussed in the literature review. Worth noting is that whilst leadership research often looks inwards to the employee/employer relationship, leadership of a school is different. A school is often the centre of the community, where it is not only employees or staff who rely on strong leadership, but also a wider community including parents; a senior leader ‘is both organizational leader and participant within a complex web of formal and informal inter-school and multi-agency partnerships . . . with different structures, professional cultures, and accountabilities’ (Hulme et al, 2021: 5). Throughout this article, when referring to leading and managing I will specify whether this relates to staff, students or others.

### Foster a shared purpose

This time of unprecedented change and anguish required a leadership response which focused efforts on ‘engaging a community in facing up to complex collective problems’ (Grint, 2010 in Wilson, 2020: 284). The collective problem was clear: students had been accessing school through virtual learning and we believed that for them to return to school after the summer break was essential; as stated in the *Together Again* document that was produced as part of our reopening, ‘we recognize the importance of having our students and staff physically present in school’ (2020). However, risk of COVID-19 infection remained, and safety needed to be the highest priority.

At the time of sharing the initial intention to reopen, we believed the community would celebrate and be grateful to have the opportunity for their children to return to school. Whilst this was the case for many, opinions garnered through an initial survey made clear there were parents who were concerned for the health of their children and did not want them to return so soon. Equally, many staff expressed that they did not feel comfortable returning to the school building. Public schools in the city were not reopening, and did not return to full in-person learning for more than 500 days from the first day of mandated lockdown, 20 March. There were those in the School X community who believed we should follow suit, whereas School X was actually closed for only approximately 140 days. Managers of the school group had made it clear we were to reopen, and many parents felt reopening was the right path to take. It was also the belief of SLT members that reopening was the right thing to do for the community for the social, emotional and academic benefit of students, the wellbeing of staff and parents, and the financial health of the school.

Wilson defines the shared purpose during the pandemic as ‘minimizing the harm to lives and livelihoods’ (2020: 285). In the case of School X, this was based on keeping students and the wider community safe from infection (minimizing the harm to lives), as well as ensuring students were effectively educated, with the secondary effect being that parents were able to return to work (minimizing the harm to livelihoods). Ardern’s government adopted ‘a precautionary, science-led approach, coupled with a willingness to act quickly and decisively’ (Wilson, 2020: 6), and members of SLT took the same approach.

Signalling our intention before the summer holidays to safely reopen after the break ensured that members of our community understood our purpose and appreciated the resoluteness of our decision, consistent with the view of Netolicky that to ensure trust in the shared purpose, ‘[i]n a time of crisis, leaders must act swiftly and with foresight’ (2020: 392), without fear of error or mistake. Leithwood et al (2020) include a shared vision as one of the leadership practices within their four domains of practice for successful school leaders (p8); for all school leaders, while ‘shared vision and moral purpose anchor . . . decisions and align operations with strategy’ (Netolicky, 2020: 391), this was never more important than in this time of unprecedented crisis.

Fostering a shared purpose required a level of influence over all stakeholders in the school community; an example of educational leadership ‘influencing others in educational settings to achieve goals’ (Connolly et al, 2019: 514): in this case, reopening the school. Bass (1990) states that one aspect of transformational leadership is providing a vision and sense of mission (p22), which supports Wilson’s element of fostering a shared purpose. By using open communication to share our vision and purpose to reopen the school, SLT was able to utilise influence from leadership, and an aspect of transformational leadership, to foster a shared purpose as ‘an overall objective for pandemic leadership’ (Wilson, 2020: 285). Our influence was demonstrated in the ‘commitment to offering the very best for our students within the unique set of circumstances we find ourselves in’ (School X, 2020d: 2).

As indicated in Figure 1, Wilson considers the ‘key leadership practices’ discussed below to be essential to build ‘trust in leadership’ (2020: 285) in leading a community through the pandemic; as Ahlström et al state, ‘[w]hen experiencing anxiety and uncertainty, trust is an important driving force to organise and deal with these challenges’ (2020: 39). Despite a number of dissenting voices in the community, through the influence of educational leaders and utilising transformational leadership, SLT sought to achieve the umbrella aim of ‘fostering a shared purpose’, or ‘building, and drawing on, a sense of shared social identity (a sense of “us-ness”)’ (Haslam et al, 2021: 49) which is ‘anchored by the trust that is built through these practices’ (Wilson, 2020: 285).

### Be led by expertise

Leithwood et al suggest that effective school leaders are ‘open-minded and ready to learn from others’ (2020: 14); this has never been more important than when leading through a global pandemic, where ‘leaders must first themselves be willing to be led by those with relevant expertise’ (Wilson, 2020: 286) in order to build trust with the wider community. This was a difficult task in School X, given its location and therefore political context; at various times in the midst of the pandemic, the then US President suggested that injecting bleach might be an option to cure COVID-19 (BBC News, 2020) and regularly mocked wearing of masks to protect ourselves, and others, from the disease (Cathey, 2020). There was a battle to make decisions for our school not only in order to keep the community safe, but also against the misinformation that was contributing to the ‘dysfunctional and ineffective pandemic leadership’ (Wilson, 2020: 286) to be found in many parts of the world.

As a leadership team, openness to science and learning from expertise was at the forefront of our decision-making process; actively seeking out that information was the first step to the reopening plan. It should be noted that, in the city, state and federal jurisdictions in the country where School X is located, there were no mandated guidelines for schools; decisions were left to districts or individual schools. As a private school, there was no district support, and the fact that the company that owns the school is spread across a number of geographical areas meant that creating standard operating procedures across all schools was impossible; we were considered by the company to be the experts on our school and our area.

After much discussion it was decided that, even without the official jurisdiction of the state (referred to here as State Y), School X would use the state school board guidance that was developed in conjunction with the state public health department (State Y Board of Education, 2020) so their expertise would influence decisions around policies and procedures; a number of other expert bodies were also consulted. These documents led to the creation of internal and external procedures including, though not exclusively:

- Arrival and dismissal procedures, including symptom screening
- Movement around school for staff, students and wider community
- The set-up of classrooms
- Lunchtime procedures
- Specialist teacher guidelines
- Virtual provision

It is also worth noting that a travel restriction was levied by the city that included compulsory quarantine for travel beyond the city (City Z, 2020); it was our decision to adhere to this, asking parents to keep children home from school after travel outside City Z’s limits.

Processes were written as temporary policies and shared through a variety of media for members of the community. Parents received regular newsletters, either written or by video, to share the information. Students received age-appropriate videos to help them understand what school would look like when they returned to the building, and to allay their fears and anxieties. Staff were given information during induction week, the two weeks leading up to students returning after the summer. Whilst these induction periods would usually be devoted to professional learning and development, in the 2020/21 school year they were dedicated to new procedures and processes.

To build trust in the community, it was necessary to show that we were following science; that we were led by the expertise available to us as a school, as supported by Wilson’s framework. Such trust ensured that the community was willing to be led by us. This was a cycle that required constant communication, along with an unwavering commitment to adherence to the procedures, no matter the circumstances. As seen below in this extract from the reopening document shared with the community, we demonstrated our commitment to being led by expertise, as well as clearly identifying the sources of our information:

*We have seen in recent weeks that the guidance can fluctuate on a weekly/daily basis, so whilst it is important for us to be clear and detailed in our plans, we must also accept the inevitability that this is an ‘ever-evolving’ situation. We must therefore strike the right balance of certainty and flexibility, based on what we know today and what is likely tomorrow. At the end of this document you will find links to the authorities who have been consulted in the creation of this guidance and on whom we will rely for further updates.*

(School X, 2020d: 2)

Designing and embedding the above processes lies within the realm of ‘carrying the responsibility for the proper functioning of a system in which others participate’ (Connolly et al, 2019: 505); they are an example of educational management, of hierarchical decisions made at the ‘top’ levels of the school and communicated with those ‘below’. ‘Proper functioning’, in this context, meant that no member of the community was infected with a potentially deadly disease – the ‘invisible rucksacks’ that James and Vince (in Connolly et al, 2019: 507) use as a metaphor for responsibility could not have been any heavier.

Though transactional leadership is often linked with task management and top-down leadership (Berkovich, 2018), in this case it was necessary to adopt this style of leadership to ensure that health and safety was top priority, perhaps even over our knowledge of teaching and learning ‘best practice’. When information about classroom layout, or arrival and dismissal, was presented to staff, this was a transaction of information, an understanding that the community was obliged to adhere to all of the rules being outlined, referring back to Avci’s (2015) point that we were purely concerned with activities that served the primary function of the organization at this time, which was students and staff being in the school building, safely and healthily.

In support of the use of transactional leadership, Hallinger (2018) determined that, whilst turning around a school from challenging circumstances, ‘successful principals tended to rely on a unitary directive leadership style focusing on establishing safety [and] order’ (2018: 15); a *unitary directive leadership style* suggesting one person (or a group acting as one) making decisions and passing them ‘down the chain’, similar to transactional leadership. Whilst Hallinger, when referring to a ‘turnaround school’, was not referring to schools reopening after a period of mandated lockdown, in these circumstances for School X the same sentiment applies. Berkovich (2018) concurs, noting that in schools with challenging contexts, transactional leadership is ‘more likely to have positive implications for their image, as perceived by staff’ (2018: 358), demonstrating that in ‘complex leadership situations’ this style is appreciated.

It would seem that in School X this approach was effective; the school building opened safely and, although tweaks were made once procedures were in place, they were generally followed and staff and students remained safe, with only 17 COVID cases in the academic year reported at the time of writing. Despite Bass’ assertion that transformational leadership is superior to transactional, he does suggest that ‘leader-follower transactions dependent on contingent reward may also work reasonably well if the leaders can provide rewards that are valued by followers’ (1990: 23); one could surmise that the reward of everyone being safe in school was valued by the community and that therefore, in this case, transactional leadership practices were successful.

### Mobilise collective effort

Fostering a shared purpose is vital as the overarching concept of this framework, while to build trust, mobilising the community in a collective effort to achieve said purpose is one of the key leadership practices required. In PM Ardern’s efforts to mobilise the New Zealand population, a ‘strong emphasis ha[d] gone to inform and educate’ (Wilson, 2020: 286) about the pandemic; at School X, a similar emphasis was placed on informing and educating about in-school practices, to reassure the community that safety was the priority. This was reflected in the induction schedule for staff, as well as in a number of communications to the wider community, including the *Together Again* document. Information included cleaning schedules and details of student bubbles, classroom layouts and visitor guidelines (School X, 2020d), as well as videos for students about health and hygiene (School X, 2020b): information that is not usually shared with the community. We maintained an unrelenting ‘commitment to regular and open dialogue’ (School X, 2020a) to create ‘the foundation for the kind of shared understanding of the nature of the problems and what needs to be done about them’ (Wilson, 2020: 286) in order to begin rallying the community towards achieving our collective shared purpose.

Informing and educating the community is important in building trust, and requires at times giving ‘hard (but credible)’ messages (Wilson, 2020: 286). Pulling no punches, as Wilson refers to it, requires honesty in messaging and communication as a leader; not to scare, but to ensure that all stakeholders understood the risks and possible outcomes of reopening the school. We opted to make it clear from the outset that, despite all the hard work and effort, there was no guarantee that students would not be exposed to, or infected with, COVID-19; in the second paragraph of the reopening document the American Academy of Pediatrics is quoted, informing the community that policies and procedures were designed to *mitigate* risk, not *eliminate* risk (School X, 2020d: 2). Parents and staff were given the difficult but truthful message that there was an element of danger in the return to school.

Remaining empathetic yet informative and sincere was, and remains, essential throughout the pandemic. It was imperative that staff understood that we heard their fears and concerns, and that they were not being disregarded; Heifitz, in Wilson (2020), calls this ‘regulating distress’ (p287), where the behaviour of the leader helps to reassure staff that despite being overwhelming, the change is necessary. This was displayed to staff through open and honest communication during the induction sessions; by explaining to staff that the need to reopen school was based on our belief that students should be in the school building, as well as for the financial security of the school (which, in turn, impacts job security). As well as having an open-door policy where staff could ask questions and air concerns, we were able to convey ‘a higher purpose and meaning . . . . whilst also conveying an empathetic appreciation of the personal impacts’ that each staff member was experiencing (Wilson, 2020: 287). We had to make it clear that we were attempting to ‘manage different tensions simultaneously’ (Bauwens et al, 2021: 3).

Wilson’s term ‘collective effort’, whilst not directly used in communications from the leadership team, was certainly conveyed in an effort to unite all stakeholders in the school community. All communications used pronouns such as ‘we’ and ‘us’ to express the community spirit needed to successfully navigate the pandemic. As was reflected across the country and the world, the school community had a variety of views as to whether the school should open, how it should open and how it should operate; parents were unafraid to share these views with SLT throughout the pandemic. With this in mind a direct call to unity was necessary, and the message below is in the opening page of the reopening plan:

*This is an undertaking without precedence, and us such our decision will never be met with universal approval. Opinions within our community will vary based on family circumstances, sources of information and our own personal experiences. This confluence of ideas and viewpoints has the ability to create division and uncertainty within our community. However, I would like to appeal to all within the [School X] community to remember what sets us apart from others, that sense of community and support that has guided us prior to and during recent changes and will continue to keep us united through the months ahead.*

(School X, 2020d: 2)

Whilst school leaders (or any leaders) rarely attract universal approval, unity was more important than ever during this time, as each person’s actions had the ability to influence the health and safety of members of the community. We were therefore asking that all stakeholders commit to the shared purpose of reopening school, and appealing for togetherness was another step in the effort to build trust.

**Figure 3.**
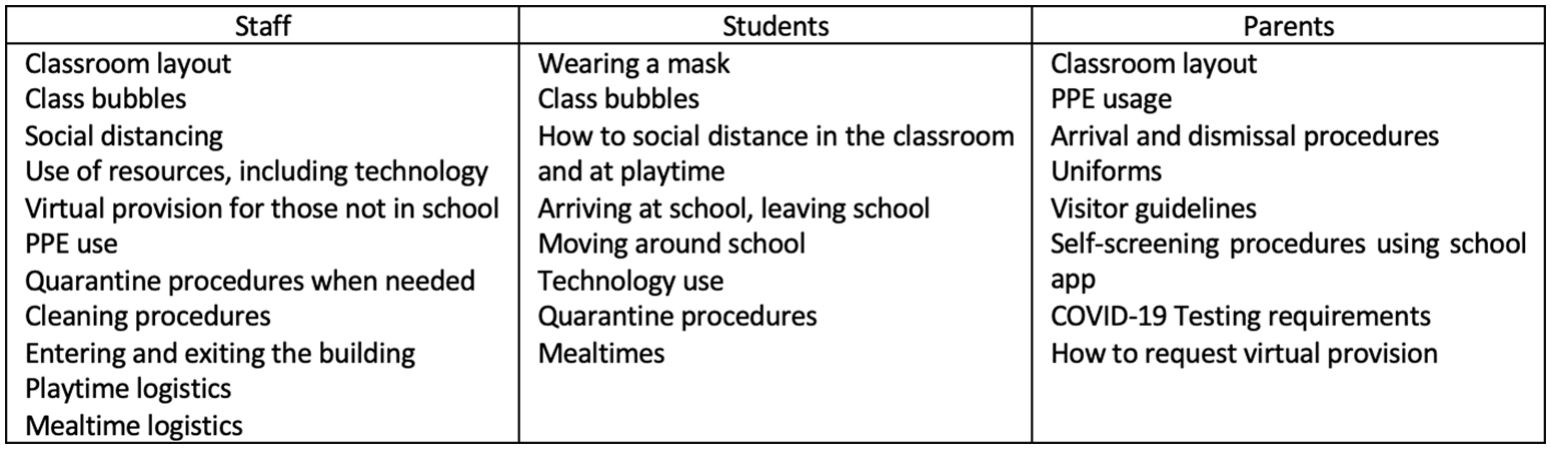
List of practicalities for various School X stakeholders.

Wilson’s framework outlines the importance of addressing practicalities (2020: 287). As for all aspects of the reopening, three groups, and their unique needs, were considered (see Figure 3 for an extensive, but not exhaustive, list).

As a leadership team, we understood the need to address these early in the communication cycle, to avoid panic and multiple questions about the same issues; Haslam and Reicher, in Wilson (2020), suggest that ‘attentiveness to such matters demonstrates a leader’s interest in, knowledge of and concern for matters that affect those they lead’ (p287) as another step to building trust. The *Together Again* document was used to address the majority of these practical concerns, as were videos referenced earlier in this article.

As with any change, decisions made are not always going to be popular or be seen as correct. Wilson (2020) outlines the need to avoid defensiveness when criticisms are levelled at leaders; separately from this, she suggests that soliciting feedback is another key practice in building trust. I would contend that these two practices should be combined. Asking for feedback and demonstrating the need to act on it can help to eliminate any inclination for defensiveness; in challenging contexts, engaging in ‘self-criticism’ and being ‘able to admit to others when they had made a mistake’ is a quality of an effective leader (Harris and Chapman, 2002: 11). To hear criticism levelled at SLT, often personally, and act upon it without defensiveness is by no means an easy feat, and it may be that we were not always successful   though showing members of the community, including staff, students and parents, that feedback is being received and listened to demonstrates Tourish’s ‘critical upwards communication’ which ‘is a critical safeguard against dysfunctional leadership’ and a key to mobilizing collective effort (in Wilson, 2020: 288). Criticism, as well as decisions that turned out to be incorrect, are par for the course in any leadership, but particularly in a challenging context such as this, ‘hence a high degree of trust will be needed, as the collective glue, to ensure that issues are addressed collectively as they arise’ (Harris and Jones, 2020: 246).

The need for trust was demonstrated after the initial reopening, as the virtual offer that was created for those students who genuinely could not enter the school building (for a variety of reasons, such as testing positive forcing a period of isolation, or being immunocompromised and thus at risk) was being used by some parents for purposes for which it was not intended, placing undue pressure and stress on teachers. For example, a number of parents were using the system to take vacations and have their children join calls from the poolside, or were using it to ‘quarantine’ at home prior to travel in order to eliminate the risk of exposure to infection which would disrupt their travel plans. Not only was this a cause of stress for the teachers and for those students still in the classroom it was also an unpleasant reminder to staff that due to quarantine requirements we were unable to leave City Z, which staff voiced was impacting negatively on wellbeing. This issue was addressed in a newsletter on 3 September 2020, just two weeks after reopening, in an effort not only to reiterate expectations with parents, but also to demonstrate to staff they were supported, thereby building trust. At this point, parents were asked to ‘apply’ for the virtual provision, with a member of SLT determining the validity of the reason for the application before giving permission; this was not a popular decision with the parent community, but one we felt was essential in order to maintain the ‘collective effort’ from staff. Equally, when ‘bubbles’ of the same classes had to be quarantined twice in a short space of time due to COVID exposure, parent and staff feedback led to a swift SLT decision to ‘unbubble’ classes: two or three classes were bubbled for timetabling purposes, particularly for playtime, to allow students to maintain and develop relationships across the year group, unbubbling isolated classes so children could only interact within their class, thus reducing possible exposure.

Much of the mobilising collective effort section of the framework fell under the guise of educational leadership, with the central theme of influence: influence to change, even when the change was unwelcome; influence to support and unite, even in times when one might disagree with decisions; influence to join together to actively work towards the shared purpose of saving lives and livelihoods. Although there were times when feedback required changes to the policies in place, the determination of our staff to ensure each child was happy, safe and learning was evident. It was clear that the community was collectively working towards the shared purpose, or ‘mutual purpose’ as Rost and Burns describe; ‘when one sees mutual purposes being forged in a relationship, that is a cue that leadership is happening’ (1993: 151).

Transformational leadership was utilised in the effort to mobilise the community; in particular, the characteristics of *charisma* and *intellectual stimulation* (Bass, 1990: 22). By consistently seeking to build trust, through empathetic yet direct messaging, through pulling no punches, through seeking feedback and acting upon it, SLT was able to provide ‘a vision and sense of mission’ that is indicative of a charismatic leader (Bass, 1990: 22). Equally, SLT exercised intellectual stimulation through sharing decision-making that was rational and, rather than being defensive in the face of criticism, acting upon it to solve problems (Bass, 1990: 22). This level of leadership influence served to intrinsically motivate the community, staff and parents, to mobilise collective effort towards the shared purpose of reopening the school.

When considering leadership *vs* management in mobilising collective effort, the possible exception is ‘addressing practicalities’ which, at first glance, falls under educational management, as it is concerned with the functioning of the organisation. However, this is an example of conflation between educational leadership and educational management, as discussed by Connolly et al (2019: 507). As they point out, the acts of management, which entail ‘co-ordinating, directing and guiding others’ to achieve a shared goal, will ‘inevitably influence others’ and are, therefore, leadership actions (Connolly et al, 2019: 507). In fact, the act of addressing these practical issues is demonstrative of a leadership team that care and understand the impact of these procedures on feelings of safety and security for the community, and the ‘significance of attending to these matters should not be underestimated’ (Wilson, 2020: 287). I would argue that, under the umbrella of mobilising collective effort, whilst addressing procedures and policies may seem to be part of the ‘control’ of management, they are in fact leadership activities that serve to build trust, and therefore influence the community to join the collective effort.

### Enable coping

In Wilson’s framework there are a number of practices specifically aimed at enabling the public to cope with the ‘challenges posed by the pandemic’ (Wilson, 2020: 288), necessary to achieve the shared purpose of minimising harm to lives and livelihoods. Whilst the first priority is to ensure the level of COVID-19 infections is low, there is also a need to mitigate the impact of social distancing and other measures on mental health; reports suggest that there has been a dramatic increase in reported cases of mental health concerns during the pandemic (Panchal and Kamal, 2021). Schools have always been the centre of their communities, but now more than ever, ‘school leaders are managing the emotional responses of others to this crisis including anxiety, frustration, loss, and anger’ (Harris and Jones, 2020: 245), an example of a ‘different contextual demand’ a leader may face (Leithwood et al, 2020: 9). Though on a smaller scale, school leaders across the world face the same issues as are faced by world leaders: enabling their communities to cope.

Our approach to ensuring the emotional health of our students was outlined in our *Together Again* document (p9), underscoring its importance to us as a school, and emphasising for the rest of the community why it should be at the forefront of their minds. Numerous policies were created to support staff and parents in planning for a range of eventualities, a key leadership practice in Wilson’s framework (2020: 288). An example of this was the School X COVID-19 Response Plan (2020d: 18), which outlined all the steps that should be taken by a parent, staff member and then school leaders in the case of a symptom or confirmed case. It aimed to cover every eventuality and permutation of events that could lead to the quarantine of students, ensuring that the school building was as safe as possible. Whilst there was no possibility to predict when quarantine could happen, this clear guide was a ‘critical tool that helps in preparing’ for the possibility; it also served ‘to build trust through providing transparency about decision making’ (Wilson, 2020: 288).

As general good practice in leadership, enabling sensemaking is a key practice to help those being led to cope with the pandemic (Wilson, 2020: 289); according to Sobral et al, ‘leaders are critically important in the context of sensemaking under crises’ (2020: 760). To minimise confusion and panic, generating a shared language was imperative in our communications with the entire community. Terms such as *bubbles*, *unbubbling*, *self-screener*, *Classroom Connect* and, most importantly, the distinction between *quarantine* and *isolation*, were explained in communications to all members of the community. This shared vernacular became commonplace in school conversations; clear communication using this language was another example of building trust amongst the community.

At the time of reopening the school, City Z was in a period of civil unrest that neither the country, nor the world, had seen in recent history (Drew, 2020); kindness seemed to be lacking in all corners of society. School X has always taught kindness, among 22 other values, through its Values curriculum, and though we were unaware at the time that enabling kindness was one of the key leadership practices from Wilson (2020), it was something we believed was essential in order for our entire community, students, staff and parents, to continue to remain mentally and emotionally healthy. While PM Ardern reassured New Zealand children that the Easter Bunny was an essential worker able to go out to work (Chappell, 2020), and medical leaders such as Dr Anthony Fauci explained to US children that Santa Claus was vaccinated so could deliver presents (Schwartz, 2020), we looked for ways to demonstrate kindness, as well as to enable others to be kind, in tangible ways within our community.

To bring back a semblance of normality, as well as to generate excitement, at special holidays such as Halloween children were encouraged to dress up and received sweet treats provided by school, breaking School X’s usual and long-standing ‘no-sugar’ policy. School celebrations continued, such as the weekly awards assembly, with immense effort put into a virtual version to ensure all students felt valued. Most impactfully, in the face of a time in education when innovation was overshadowed by ‘getting the job done’ because we needed to accept that ‘staff are living through the crisis as well and incorporate some consistency in order to help negate staff anxiety’(Hudson et al, 2021: 22), we realised the importance of building a culture of kindness and acceptance; Black History Month, American, Asian and Pacific Islander Month, and Pride Month were all this academic year embedded into the curriculum for the first time. Many of these initiatives were not generated by SLT, but by staff, parents and students, in response to current events; they demonstrate the importance placed on kindness in School X’s community, which was appreciated in trying times, and served to build trust within, and in, the school.

Wilson’s final key leadership practice is developing creative responses (2020: 289); the development of a ‘new normal’ has forced many industries, including education, to think ingeniously to solve challenging problems. The quick turnaround to virtual teaching for all schools going into lockdown was an initial ‘creative response’; for School X, however, reopening posed further problems. Our belief was that all children should be in school, that this was the best place for their emotional health as well as their academic success, although this view was not met with universal approval. As indicated in response to an informal survey, many families were unwilling to send their children to school (School X, 2020c). With public schools in the city remaining closed and offering a virtual alternative, it was apparent that in order to maintain the financial health of School X it would be necessary to find a solution that would appeal to these families. From this, Classroom Connect was born: an option whereby students could join their class, and follow the timetable, from home via Microsoft Teams. Consistent with our commitment to honest and open communication, leaders were very clear about this option:

*While we know nothing can replace the experience students get from being in a real classroom, we want to ensure that everyone who wants to be a student at [School X] is able to do so, regardless of their location or personal/family situation.*

(School X, 2020d: 14)

Classroom Connect required hardware and infrastructure in the classroom, as well as staff commitment and creativity to ‘make it work’. It was developed as a creative response to two problems: the first, families who wanted a quality education for their child/ren but were unwilling to send their children to school, thereby building trust with the parental community that we heard their concerns and were willing to find solutions. Secondly, the potential loss of these families that could severely impact the financial health of School X and therefore potentially create redundancies, a reality at other schools in the group. Though a difficult decision to take, and one that as educators we did not feel was in the best interests of the students and teachers, it demonstrated to parents and staff that there was a commitment from leaders to take a pragmatic approach in order to minimise harm to lives and livelihoods, building trust that ‘leadership is committed to the shared purpose’ (Wilson, 2020: 290).

Enabling community members to cope does seem as though it lends itself to educational leadership, leading others to a shared purpose of minimising risk to lives and livelihoods; if we return to the idea of leadership being ‘influence to achieve organizational goals’ (James et al, 2020: 2),then enabling the staff and wider community to cope, emotionally and practically, was essential to achieving the goals of the organisation. Many of the actions in this part of the framework, however, also fell under the guise of educational responsibility, driven by ‘an internal sense of obligation’ (Connolly et al, 2019: 513), where obligation ‘is not an action’ (2019: 513), but underpins actions, as it did our actions to assist the community to cope.

Transformational leadership was required through much of this key practice. With creative problem-solving such as introducing virtual provision (according to Bass (1990), an example of intellectual stimulation) as well as communicating expectations and providing the ability to make sense of the situation (which is an example of inspiration), the leadership team at School X were able to build trust, creating relationships with the community that encouraged them to ‘make more of a contribution to the organization’ (Bass, 1990: 22). Farhan, in her analysis of Canadian Prime Minister Trudeau’s response to COVID, described a similar leadership practice in the third stage of leadership, *Challenging*; she suggests that this stage ‘highlights the role of transformational leadership in encouraging . . . . people to show their creativity’ (2021: 6). However, there were times when transactional leadership was required; for example, using the contingent reward of no job losses to explain to staff why the inconvenience and challenge of virtual provision was a ‘necessary evil’.

## Conclusion

School leaders . . . are defined by their determination, their hope, and their unshakable belief that whatever happens, whatever the cost, whatever the scale of the challenge, they will continue to do everything in their power to safeguard the learning of all young people.

(Harris and Jones, 2020: 246)

Without doubt, the leaders of School X were driven by concern for the education, physical and mental health of the young people in our care. In school leadership, however, building ‘social trust’, of the ‘interconnectedness of home, school and the community’ (Harris and Chapman, 2002: 12), is paramount, and we very much understood the need to build trust within our community. Whilst the Wilson framework was not available when we were reopening the school, upon reflection and analysis of our efforts, it seems that the New Zealand government and SLT were on similar paths with the same shared purpose, minimising the impact of the pandemic upon our respective communities.

It is also clear that, especially in times of crisis, educational management and educational leadership need to be employed as appropriate; management should not be denigrated in order to ennoble leadership (Rost and Burns, 1993: 140). While management can create connotations of top-down hierarchy, there are times when it is essential that the responsibility lies with one person or group; when certain activities are not completed (not complying with quarantine guidelines, for instance), there can be ‘crucial implications’ (Connolly et al, 2019: 508). Educational management is essential to the functioning of an organisation. This is also true of transactional leadership, which had to be utilised at several points during the reopening; although transformational leadership is ‘widely advocated as an appropriate model of educational leadership’ (Connolly et al, 2019: 513), there are times when, as Berkovich (2018) contends, employing aspects of transactional leadership is likely to have a positive influence on staff perception of a leader’s image, ‘suggesting that in complex leadership situations, individuals value a more hands-on style’ (Berkovich, 2018: 358).

Although management and transactional leadership were essential during this period, so were educational leadership and transformational leadership. Central to leadership is influence. As a leadership group, we were aiming to move or motivate people ‘to think/feel/act in some way’ (Connolly et al, 2019: 512); in this case, to share our purpose, as per the Wilson framework. At most points throughout this vignette, leaders of School X were relying on the intrinsic motivation of staff to believe in the changes being made, to act in a manner that suggested they were ‘on board’, and to make the difficult circumstances work for the community, all in an effort to ‘minimize harm to lives and livelihoods’ (Wilson, 2020: 285). It is pertinent to note that transactional leadership does not always equate to educational management; Bass (in Rost and Burns, 1993: 132) explained that it can be an example of leadership, which I would argue in this case it was. Transactional leadership was used to influence staff into accepting the difficulties and to ‘make it work’ in order to keep the school open, influencing their actions in order to ‘achieve organizational goals’.

Context and circumstance were the influencing factors throughout this vignette. At times, usual leadership practices were abandoned as new paths were navigated; as noted by Harris et al (2020), ‘in such disruptive times, school leaders cannot emulate the leadership practices they witnessed or enjoyed in a period of stability, continuity, and relative calm’ (Harris and Jones, 2020: 246). It was necessary to adapt and change according to the situation, or aspect of reopening; this was expected by the staff and the wider community; while ‘expectations of principals to be transformational leaders are present in all circumstances, . . . their expectations of principals to adopt a transactional . . . style is context-dependent’ (Berkovich, 2018: 358).

‘[L]eading in the context of a pandemic is no easy feat’ says Wilson (2020: 290), and the weight of responsibility, whether a heavy backpack or a weighted crown, is inescapable. The leadership team of School X worked tirelessly, as every school leadership team around the world has, to ensure the safety, health and wellbeing of the community and the academic progress of each student in the school. At the time of writing, the school is one week from closing for the academic year, and from the reactions of the community, it is clear that we have made ‘a significant difference’ by ‘doing quite a few things well’ (Wilson, 2020: 290). Though I am sure that, given time to reflect, there may well be things the SLT of School X will believe they might have done differently, using the Wilson framework as a lens through which to view events I argue that, if PM Ardern’s work is a measure of success, by effectively utilizing educational leadership and management, as well as transactional and transformational leadership, and underpinned by our educational responsibility, we achieved our purpose of minimizing the risk to our community’s lives and livelihoods, and led the community effectively through the reopening of the school.
